# Wnt Signaling Mediates the Aging-Induced Differentiation Impairment of Intestinal Stem Cells

**DOI:** 10.1007/s12015-019-09880-9

**Published:** 2019-02-21

**Authors:** Hui Cui, Duozhuang Tang, George B. Garside, Ting Zeng, Yiting Wang, Zhendong Tao, Liu Zhang, Si Tao

**Affiliations:** 1 Jiangxi Key Laboratory of Clinical and Translational Cancer Research, Department of Oncology, The Second Affiliated Hospital of Nanchang University, Min-De Road. 1, Nanchang City, 330006 Jiangxi Province China; 2 Department of Oncology, The Second Affiliated Hospital of Nanchang University, Min-De Road. 1, Nanchang City, 330006 Jiangxi Province China; 3 Department of Hematology, The Second Affiliated Hospital of Nanchang University, Jiangxi, China; 4 Leibniz Institute on Aging - Fritz Lipmann Institute (FLI), Jena, Germany; 5 Department of Medical Laboratory Medicine, Jiangxi Province Hospital of Integrated Chinese & Western Medicine, Jiangxi, China; 6 Intensive Care Unit, Peking University People′s Hospital, Beijing, China

**Keywords:** Aging, Wnt signaling, Intestinal stem cells, Differentiation, R-spondin-1

## Abstract

Stem cell aging underlies aging-associated disorders, such as steeply increased incidences of tumors and impaired regeneration capacity upon stress. However, whether and how the intestinal stem cells age remains largely unknown. Here we show that intestinal stem cells derived from 24-month-old mice hardly form typical organoids with crypt-villus structures, but rather mainly form big, rounded cysts devoid of differentiated cell types, which mimics the culturing of heterozygous APC-deficient cells from the *APC*^*min*^ mouse line. Further analysis showed that cultured crypts derived from aged mice exhibited reduced expression levels of differentiation genes and higher expression of Wnt target genes. Lowering the concentration of R-spondin-1 in the culture system significantly reduced formation of rounded cysts, accompanied by an increased formation of organoids from crypts derived from old mice. We are the first to uncover that intestinal stem cells derived from old mice harbor significant deficiency in differentiation that can be partially rescued through a reduction in R-spondin-1 exposure. This could be highly relevant to intestinal tumor development and the reduced regeneration potential observed in the aged population. Our study provides the first experimental evidence that an over-responsiveness to Wnt/beta-catenin signaling of aged intestinal stem cells mediates the aging-induced deficiency in differentiation, and could serve as a potential target to ameliorate aging-associated intestinal pathologies.

**Electronic supplementary material:**

The online version of this article (10.1007/s12015-019-09880-9) contains supplementary material, which is available to authorized users.

## Introduction

Aging-associated intestinal pathologies are characterized by increased incidence of malignances and poor tolerances to stress, such as radiotherapy, chemotherapy and infection, which often cause severe complications in aged people. Intestinal stem cells (ISCs) are the driving force of intestinal homeostasis and regeneration [1, 2]. Functional abnormalities of ISCs underlie multiple intestinal diseases. First, ISCs were identified as the cell of origin in intestinal tumors which often exhibit high proliferative activities [3–8]. Second, radiation and cytotoxic therapies cause massive loss of ISCs and epithelial cells, which results in complications such as infections and diarrhea. After the acute phase of cell loss, the remaining ISCs rebuild the stem cell pool and the intestinal epithelium via proliferation and differentiation [9]. Taken together, the maintenance of intestinal homeostasis relies on balanced self-renewal, proliferation and differentiation of ISCs, which play essential roles in intestinal tumor development and regeneration after injury. However, little is known about how the core functions of ISCs alter with time, and whether their homeostatic balance is disturbed during aging.

Wnt/beta-catenin signaling pathway is a vital force to promote self-renewal and proliferation of ISCs [10–13]. Numerous studies have suggested that over-activation of Wnt signaling pathway is indispensable to intestinal tumor development [14–17]. Of note, recent studies have uncovered critical roles of Wnt signaling pathway in stem cell aging in other systems, such as the hematopoietic system and the skeletal muscle system [18, 19]. Up-regulation of Wnt signaling during aging leads to impairment of muscle stem cell function and increases fibrosis [19]. Previously, we have identified Wnt signaling as a key mediator of ISC fate decision in premature aged mice, but the function of ISCs from aged mice was not studied [20]. Recently, it was reported that aged crypts exhibited reduced regenerative potential as a result of decreased niche (Paneth/mesenchyme) secretion of Wnt signals [21]. However, the relative activities of proliferation and differentiation of ISCs of aged mice remain to be illuminated. It is known that cell proliferation and differentiation are intimately coupled in intestinal cells, and these processes are tightly regulated by Wnt/beta-catenin signaling pathway [22–24]. Elevated Wnt signaling activity constitutes a dominant switch between proliferation and differentiation, which is an essential event in the early stages of intestinal tumorigenesis [24]. Whether the Wnt signaling pathway would mediate a switch between proliferation and differentiation of ISCs during aging remains largely unknown.

In this study, we uncovered an unexpected deficiency in differentiation of ISCs from aged mice, using the 3D matrigel-based culture system, an accepted ex vivo assay that is reflective of ISC function in vivo. Astonishingly, ISCs from old mice mainly form rounded cysts devoid of differentiated cell types, distinctly different from the ISCs of young mice that mainly formed typical differentiated organoids. Furthermore, reducing exposure to the Wnt agonist R-spondin-1 in the culture system partially restored the deficiency in differentiation of ISCs from old mice. Our study provides first experimental evidence that ISCs derived from old mice harbor significant deficiency in differentiation, which can be partially reversed by reducing exposure to Wnt signaling. These findings could be highly relevant to aging-associated intestinal pathologies, such as tumor formation and impaired regeneration capacity after damage. Our study gives new insight in understanding the mechanisms underlying ISC aging, and offers hope to find potential ways to retard the above-mentioned aging-associated intestinal pathologies.

## Materials and Methods

### Mice

C57BL/6J mice were obtained from Hunan SJA Laboratory Animal Co. Ltd. (Hunan, China) and maintained in the animal facilities of Nanchang Royo Biotech under pathogen-free conditions on a 12-h light/12-h dark cycle. All mouse experiments were approved by the Animal Experimental Ethical Inspection of Nanchang Royo Biotech Co. Ltd. (RYEI20170430–1). 2 months old mice were used as young mice, and 24 months old mice were used as old mice.

### Crypt Culture

Crypt culture was performed as previously described [20] . Briefly, isolated crypts were resuspended in cold matrigel (BD) containing Y27632 (Abcam), hR-spondin-1 (PeproTech), mEGF (PeproTech), hNoggin (PeproTech), and penicillin/streptomycin, and plated in 24-well plates at a density of 200 crypts, 50 μl per well. The plate was incubated at 37 °C for 5 min, and then 500 μl of advanced DMEM/F 12 medium (Invitrogen) containing B27 (Invitrogen), N2 supplement (Invitrogen) and 1.25 mM N-acetylcystein was added to each well to cover the matrigel. In the culture system final concentrations of the following components were R-spondin-1 1 μg/ml, mEGF 50 ng/ml, and Noggin 100 ng/ml. Y27632 10 μM was included in the first 3 days after seeding. Final concentration of mWnt3a (R&D Systems) was 100 ng/ml. The medium containing growth factors was changed every 3 days, and crypts were passaged after 10 days of primary culture. Growth factors were added every other day and culture medium was changed every 4 days.

#### RNA Isolation and cDNA Synthesis

Total RNA was isolated from cultured crypts by using RNApure Tissue Kit (CWbiotech) following the manufacturer’s instructions. Reverse transcriptions were performed to synthesize first-strand DNA by using TransScript-Uni One-Step gDNA Removal and cDNA Synthesis SuperMix (TransGen Biotech).

#### Quantitative Real-Time PCR (qRT-PCR)

qRT-PCR was performed with an ABI 7900 Real-Time PCR system (Applied Biosystems). TransStart Tip Green qPCR SuperMix (TransGen Biotech) was used. Primer sets for the detection of single genes are listed in Supplementary Table S1. mRNA expression of genes was normalized to beta-actin in each sample.

#### Statistics

GraphPad Prism 7.0 software was used for statistical analysis. The unpaired two-tailed Student’s *t* test and two-way ANOVA were used to calculate *p*-values for two-group datasets and four-group datasets, respectively.

## Results and Discussion

### ISCs from Aged Mice Exhibit Significant Deficiency in Differentiation in Culture

**Fig. 1:**
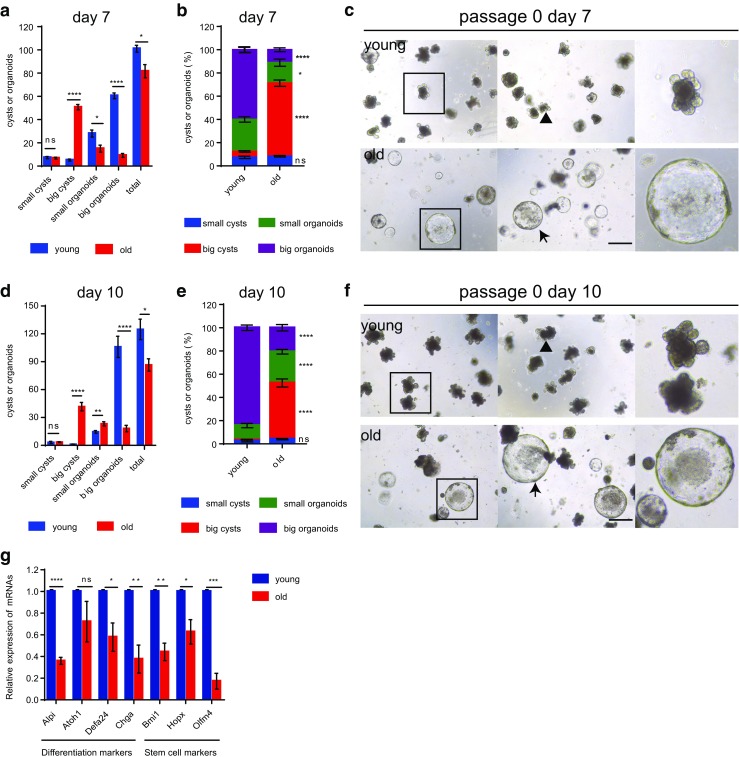
**ISCs from aged mice exhibit significant deficiency in differentiation in primary culture.** Freshly isolated crypts from 2 months old (young) and 24 months old (old) mice were plated at a density of 200 crypts per well (results show data from one representative experiment out of 2 independent experiments; *n* = 3 mice per group). (**a**, **b**) Absolute number (**a**) and percentage (**b**) of grown out structures on day 7 after plating. **c** Representative pictures of indicated groups on day 7 after plating. **d,e** Absolute number (**d**) and percentage (**e**) of grown out structures on day 10 after plating. **f** Representative pictures of indicated groups on day 10 after plating. Small cysts: diameters ≤70 μm; big cysts: diameters >70 μm; small organoids: with crypt-villus architectures, budding number ≤ 3 (for **a** and **b**) and budding number ≤ 5 (for **d** and **e**); big organoids: with crypt-villus architectures, budding number > 3 (for **a** and **b**) and budding number > 5 (for **d** and **e**). Arrow heads indicate typical organoids; arrows indicate typical big cysts. **g** mRNA expression of indicated genes in crypts cultured for 7 days (*n* = 3 independent experiments). mRNA expression of genes was normalized to beta-actin with the expression level of each gene in young crypts set to 1. Data are displayed as mean ± SEM. *, *P* < 0.05; **, *P* < 0.01; ****, *P* < 0.0001. ns, not significant. Unpaired two tailed Student’s *t* test was used. Scale bar: 200 μm

**Fig. 2:**
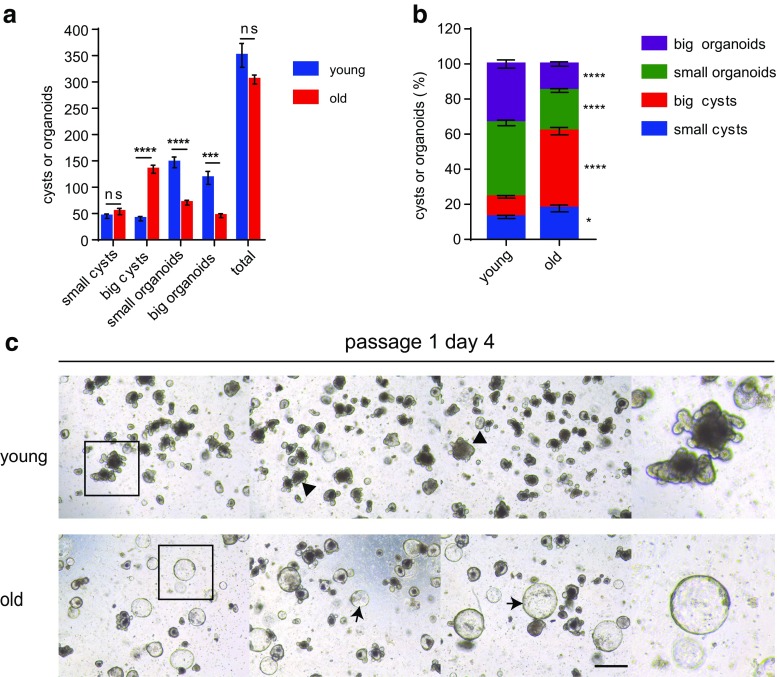
**ISCs from aged mice exhibit significant deficiency in differentiation in subculture. a**-**c** Cultured crypts were dissociated and passaged on day 14 after primary plating. **a**,**b** Absolute number (**a**) and percentage (**b**) of grown out structures. **c** Representative pictures of indicated groups on day 4 after secondary plating. Small cysts: diameters ≤70 μm; big cysts: diameters >70 μm; small organoids: with crypt-villus architectures, budding number ≤ 3; big organoids: with crypt-villus architectures, budding number > 3. Arrow heads indicate typical organoids; arrows indicate typical big cysts. Data are displayed as mean ± SEM. *, *P* < 0.05; ***, *P* < 0.001; ****, *P* < 0.0001. ns, not significant. Unpaired two tailed Student’s *t* test was used. Scale bar: 200 μm

In a 3D matrigel culture system containing growth factors including Wnt agonist R-spondin-1, EGF, and Noggin, freshly isolated crypts from mouse whole small intestine autonomously grow into organoids, recapitulating the normal gut epithelium. An organoid contains crypt-like structures with de novo generated stem cells. These in turn differentiate to produce Paneth cells within the crypt domain, and transit-amplifying cells at the upper part of these crypts, which feed into villus-like luminal domains containing post-mitotic enterocytes and goblet cells. The organoids can be dissociated into single cells and replated to form new organoids for several passages. Thus, the formation of a crypt-villus axis in the culture system reflects the proliferation as well as the differentiation activity of the plated ISCs [25] . A previous study has shown that duodenal crypts from aged mice exhibit reduced rate of organoid formation after the third passage [21]. We sought to further study the function of old ISCs after primary plating using the matrigel culture system, which may more directly reflect the potential of proliferating and differentiation. To this end, isolated crypts from the whole small intestine, an enriched compartment of ISCs, from young and old wild type C57BL/6 mice, were plated. The efficiency of outgrowth and budding was compared to reflect proliferation and differentiation of the plated ISCs. ISCs from old mice showed a slight decrease in the number of total outgrowth compared to young mice (Fig. 1a) on day 7 after plating. We noted 3 kinds of typical outgrowth of the cultured crypts: small cysts (diameter ≤ 70 μm, without any budding); big cysts (diameter > 70 μm, without any budding, indicating proliferating without differentiation); organoids (with buddings, indicating balanced proliferation and differentiation). In line with other studies, crypts derived from young mice formed normal organoids (Fig. 1a–f). In total, on day 7 after plating, organoids with crypt-villus architectures constitute more than 85% (87.6 ± 0.5%) of the grown out structures (Fig. 1b,c). The culture of young crypts continuously develop more organoids afterwards and on day 10 after plating more than 95% (96.5 ± 0.8%) of the grown out structures were organoids in which more than 80% (84.2 ± 2.3%) were big organoids having more than 5 buds (Fig. 1d–f). Strikingly, ISCs derived from 24 months old mice formed mainly rounded cysts devoid of differentiated cell types: on day 7, more than 60% (62.5 ± 2.7%) of grown out structures were big rounded cysts (Fig. 1b, c), and even on day 10, still more than 45% (47.1 ± 3.5%) of grown out structures were rounded cysts some of which grew considerably, to sizes bigger than 300 μm in diameter (Fig. 1e, f). Furthermore, the absolute number and percentage of the big organoids (having more than 3 buds on day 7, and having more than 5 buds on day 10) deriving from young crypts were significantly higher than from the old crypts (absolute number 60.5 ± 2.5 in young versus 9.2 ± 1.7 in old; percentage 60.0 ± 2.3% in young versus 11.4 ± 1.7% in old on day7; absolute number 106.0 ± 11.4 in young versus 18.2 ± 3.4 in old; percentage 84.2 ± 2.3% in young versus 20.7 ± 2.7% in old on day 10). The same morphology of rounded cysts can be seen upon culturing APC-deficient cells from *APC*^*min*^ mice [26]. These data indicate a deficiency in differentiation of ISCs derived from old ISCs. To decipher the differentiation deficiency phenotype of old ISCs, we further examined expression level of differentiation markers of the cultured crypts, including Alpi (enterocytes), Atoh1 (secretory lineage), Defa24 (Paneth cells), and Chga (enteroendocrine cells). The qPCR analysis revealed significant down-regulation of Alpi, Defa24 and Chga, with a reduced but non-significant difference in Atoh1 expression (Fig. 1g). Stem cell markers, including Olfm4, Bmi1 and Hopx [22], were down-regulated in old crypts (Fig. 1g). These results further prove the differentiation deficiency of ISCs derived from aged mice. Similar phenotypes were observed on the subsequent passage of crypts (Fig. 2a–c). Tumors usually harbor strong proliferative activities with a deficiency in differentiation. The phenomenon that ISCs derived from aged mice phenocopies APC-deficient cells from *APC*^*min*^ adenomas in culture suggests a potential similar property like APC-deficient cells which harbor imbalanced proliferation and differentiation in aged ISCs.

### Reduction in R-Spondin-1 Exposure Ameliorates the Aging-Induced Deficiency in Differentiation of ISCs

**Fig. 3:**
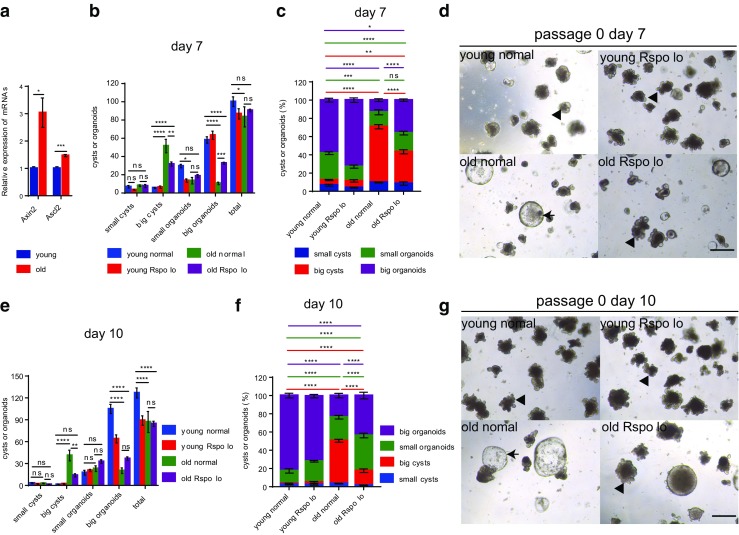
**Reduction in R-spondin-1 exposure during primary culture ameliorates the aging-induced deficiency in differentiation of ISCs. a** mRNA expression of Wnt target genes in crypts derived from 2 months old (young) and 24 months old (old) mice after culturing for 7 days. mRNA expression of genes was normalized to beta-actin with the expression level of each gene in young crypts set to 1 (n = 3 independent experiments). **b**-**g** Freshly isolated crypts from 2 months old (young) and 24 months old (old) mice were plated at a density of 200 crypts per well. Concentration of R-spondin-1was reduced to 1/3 of the normal level (330 ng/ml) in half of the wells (results show data from one representative experiment out of 2 independent experiments; n = 3 mice per group). **b**,**c** Absolute number (**b**) and percentage (**c**) of grown out structures on day 7 after primary plating. **d** Representative pictures of indicated groups on day 7 after plating. e,**f** Absolute number (**e**) and percentage (**f**) of grown out structures on day 10 after plating. **g** Representative pictures of indicated groups on day 10 after plating. Young normal: ISCs derived from young mice culturing in normal concentration of R-spondin-1; young Rspo lo: ISCs derived from young mice culturing in 1/3 concentration of R-spondin-1; old normal: ISCs derived from old mice culturing in normal concentration of R-spondin-1; old Rspo lo: ISCs derived from old mice culturing in 1/3 concentration of R-spondin-1;Small cysts: diametres≤70 μm; big cysts: diameters>70 μm; small organoids: with crypt-villus architectures, budding number ≤ 3 (for **b** and **c**) and budding number ≤ 5 (for **e** and **f**); big organoids: with crypt-villus architectures, budding number > 3 (for **b** and **c**) and budding number > 5 (for **e** and **f**). Arrow heads indicate typical organoids; arrows indicate typical big cysts. Data are displayed as mean ± SEM. *, *P* < 0.05; **, *P* < 0.01; ***, P < 0.001; ****, *P* < 0.0001. ns, not significant. Unpaired two-tailed Student’s *t* test was used for **a**. Two-way ANOVA analysis was used for **b**,**c**,**e**,**f**. Scale bar: 200 μm

**Fig. 4:**
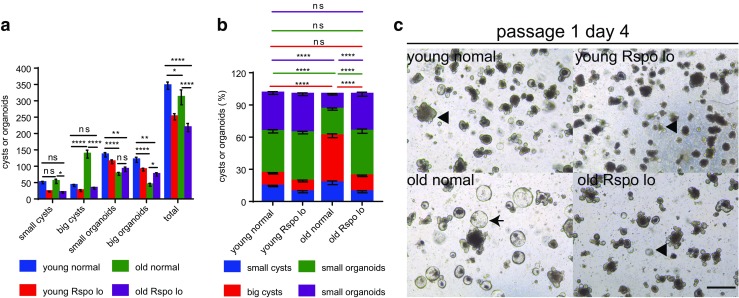
**Reduction in R-spondin-1 exposure during subculture ameliorates the aging-induced deficiency in differentiation of ISCs. a**-**c**) Cultured crypts were dissociated and passaged on day 14 after primary plating. **a**,**b** Absolute number (**a**) and percentage (**b**) of grown out structures. **c** Representative pictures of indicated groups on day 4 after secondary plating. Young normal: ISCs derived from young mice culturing in normal concentration of R-spondin-1; young Rspo lo: ISCs derived from young mice culturing in 1/3 concentration of R-spondin-1; old normal: ISCs derived from old mice culturing in normal concentration of R-spondin-1; old Rspo lo: ISCs derived from old mice culturing in 1/3 concentration of R-spondin-1; Small cysts: diametres≤70 μm; big cysts: diameters>70 μm; small organoids: with crypt-villus architectures, budding number ≤ 3; big organoids: with crypt-villus architectures, budding number > 3. Arrow heads indicate typical organoids; arrows indicate typical big cysts. Data are displayed as mean ± SEM. *, P < 0.05; **, P < 0.01; ****, P < 0.0001. ns, not significant. Two-way ANOVA analysis was used. Scale bar: 200 μm

It has been reported that Paneth cells and the mesenchyme secrete less Wnt ligands during aging, resulting in a decrease in exogenous Wnt, leading to lower Wnt signaling activity in ISCs in vivo and their reduced regenerative potential [21]. Since our study showed that culturing ISCs from aged mice mimics culturing of APC-deficient cells from *APC*^*min*^ mice, which exhibit over-activated Wnt signaling, we inferred that old ISCs might be more sensitive to R-spondin-1 induction and that normal levels of R-spondin-1 might result in over-activation of the Wnt pathway. To test this hypothesis, we examined the expression levels of Wnt target genes, Axin2 and Ascl2 in cultured crypts. Axin2 is a bona fide Wnt target gene and negative Wnt regulator induced by activation of the Wnt pathway [27]. Ascl2 is another Wnt target gene as well as a master regulator of intestinal stem cell identity, whose overexpression induces crypt hyperplasia [12, 28]. Indeed, our results showed that cultured crypts derived from aged mice exhibited significantly higher levels of Axin2 and Ascl2 (Fig. 3a). We then sought to rescue the differentiation blockage of old ISCs by reducing Wnt activity. In the culture system, the Wnt agonist R-spondin-1 potently amplifies Wnt responses, we therefore lowered the supply of R-spondin-1 to 1/3 of the normal concentration (Rspo lo) (330 ng/ml). As expected, a reduction in R-spondin-1 exposure resulted in lower expression levels of Axin2 and Ascl2 (Fig.S1). Interestingly, lowering R-spondin-1 in the culture system led to a significant reduction of big-cyst formation and an induction of organoid formation in crypts derived from aged mice (Fig. 3b–g). On day 7 after plating, both absolute number and the percentage of big cysts of the grown out structures derived from old mice decreased significantly in Rspo lo condition (absolute number 51.5 + 7.3 and percentage 61.1 + 2.3% in normal condition versus absolute number 31.7 + 2.4 and percentage 34.9 + 2.2% in Rspo lo condition) (Fig. 3b, c). Big organoid formation of the crypts derived from aged mice was also significantly improved (constituting 13.3 ± 1.5% of the grown out structures in normal R-spondin-1 condition versus 36.1 ± 1.3% in Rspo lo condition, and the absolute number increased from 10.5 ± 1.3 to 32.7 ± 1.1 in Rspo lo condition) (Fig. 3b, c). On day 10 after plating, reducing R-spondin-1 exposure still led to a significantly decreased formation of big cysts (absolute number 41 + 7.3 and percentage 47.0 + 1.6% in normal condition versus absolute number 13.8 + 2.2 and percentage 16.1 + 2.0% in Rspo lo condition) (Fig. 3e, f), and a significantly increased percentage of big organoids of the grown out structures (23.8 ± 2.4% in normal condition versus 43.8 ± 3.6% in Rspo lo condition) derived from old mice (Fig. 3f). Lowering R-spondin-1 concentration also led to a trend of increase in absolute number of big organoids derived from old crypts (20.2 ± 3.9 in normal condition versus 36.7 ± 2.5 in Rspo lo condition), although the difference was not significant (Fig. 3e). In general, lowering Wnt activity partially rejuvenated old ISCs by reversing the deficiency in differentiation, which was further highlighted in the passaged crypts (Fig. 4a–c). These results indicate that ISCs of aged mice exhibit an over-responsiveness to Wnt signaling that impairs their ability to differentiate, which could prime the aged intestine for further neoplastic processes leading to elevated tumor formation risk.

We further tested the effect of up-regulation of Wnt signaling on old ISCs. Addition of Wnt3a led to increased Wnt activity in the cultured crypts as shown by up-regulated expression of Axin2 and Ascl2 (Fig. S2A), and an elevated absolute number of big cysts, though the difference in the constituent ratio was not significant (Fig.S2B-D). The overall outgrowth also had a trend to increase, though again, not significantly (Fig.S2B). These results indicate that elevated activity of Wnt signaling further blocks differentiation of cultured ISCs, which was in line with several previous studies in which addition of Wnt3a caused the typical crypt-villus architecture to change into rounded cysts devoid of differentiated cell types [26, 29, 30].

In summary, the current study revealed that ISCs derived from old mice harbor a significant deficiency in differentiation that can be partially rescued through a reduction in R-spondin-1 exposure. This could be highly relevant to intestinal tumor development and the reduced regeneration potential observed in the aged population. Our study provides the first experimental evidence that an over-responsiveness to Wnt/beta-catenin signaling of aged intestinal stem cells mediates the aging-induced deficiency in differentiation, and could serve as a potential target to ameliorate aging-associated intestinal pathologies.

Previously, Nalapareddy et al. have reported a decrease in niche Wnt signal mainly resulting from reduced secretion of Wnts by Paneth cells and the mesenchyme in aged mice, which led to lower Wnt signaling levels in ISCs in vivo and reduced their regenerative potential. They described that the number of lobes or buds per crypt from aged intestine was lower after 3 passages in culture. The addition of Wnt3a rescued the phenotype after 3 passages. Our study showed that old ISCs formed a large proportion of big cysts with no budding, and formed significantly less differentiated, big organoids. Our study stands in agreement with their study in respect to the comparison between the number of lobes or buds per crypt.

Of note, Nalapareddy et al. only used duodenal (proximal) crypts in culture, while we tested the potential of crypts derived from the whole intestine. Multiple studies have shown that distal intestine has a higher potential to form adenomas [31–33], and the formation of adenomas was closely related to impairment of differentiation. Therefore it is highly possible that the deficiency of differentiation of the old crypts was less obvious in the Nalapareddy study where only duodenal (proximal) crypts were used for experiments.

Furthermore, our study suggested that ISCs from aged mice showed a higher sensitivity to sufficient Wnt signals supplied in the culture system which reflects an intrinsic over-responsiveness to Wnt signaling. Incidences of intestinal tumors increase sharply upon aging, with aberrant expression of APC along with other components of the beta–catenin destruction complex resulting in hyperactive Wnt signaling [23, 34–36]. This has been identified as a driving force for the development of intestinal tumors, which accumulate with age [23, 34–36]. It is tempting to speculate that the increased intrinsic over-responsiveness to Wnt signals in old ISCs could result from an altered epigenetic or genetic profile, which could be therapeutically targetable and reversed. Our study reveals an intrinsic and abnormal over-responsiveness of old ISCs to Wnt signaling, which we hope will shed new insight into the field of ISC aging.

## Electronic supplementary material

ESM 1 (DOCX 13 kb)

Fig. S1 **Reduction in R-spondin-1 exposure lowers Wnt signaling activity in cultured ISCs.** Freshly isolated crypts from 24 months old mice were plated at a density of 200 crypts per well. mRNA expression of Wnt target genes was analyzed in crypts cultured for 7 days. mRNA expression of genes was normalized to beta-actin with the expression level of each gene in crypts cultured under normal condition set to 1 (*n* = 3 independent experiments). Data are displayed as mean ± SEM. *, *P* < 0.05; **, *P* < 0.01; ****, *P* < 0.0001. ns, not significant. Unpaired two tailed Student’s *t* test was used. (PDF 837 kb)

Fig. S2 **The effect of addition of Wnt3a on old ISCs during primary culture.** Freshly isolated crypts from 24 months old mice were plated at a density of 200 crypts per well. Wnt3a was added at a concentration of 100 ng/ml. (A) mRNA expression of Wnt target genes was analyzed in crypts cultured for 7 days. mRNA expression of genes was normalized to beta-actin with the expression level of each gene in crypts cultured under normal condition set to 1 (*n* = 3 independent experiments). (B-D) Absolute number (B), percentage (C) of grown out structures, and representative pictures (D) on day 7 after primary plating in indicated groups. Results show data from one representative experiment out of 2 independent experiments; n = 3 mice per group. Small cysts: diametres≤70 μm; big cysts: diameters>70 μm; small organoids: with crypt-villus architectures, budding number ≤ 3; big organoids: with crypt-villus architectures, budding number > 3. Data are displayed as mean ± SEM. *, *P* < 0.05; **, *P* < 0.01; ***, *P* < 0.001; ****, *P* < 0.0001. ns, not significant. Unpaired two tailed Student’s *t* test was used analysis was used. Scale bar: 200 μm. (PDF 11363 kb)
