# Supplementary material for: Wnt Signaling Mediates the Aging-Induced Differentiation Impairment of Intestinal Stem Cells
Source: Stem Cell Rev. 2019 Feb 21;15(3):448–55. doi: 10.1007/s12015-019-09880-9 (PMC6534527; doi:10.1007/s12015-019-09880-9)
Supplement: Supplementary file 1 — (DOCX 13 kb) [file 12015_2019_9880_MOESM1_ESM.docx]

**Supplementary Table S1**

| **Gene** | **Forward Primers** | **Reverse Primers** |
| --- | --- | --- |
| β-actin | CTAAGGCCAACCGTGAAAAG | ACCAGAGGCATACAGGGACA |
| Axin2 | GAGAGTGAGCGGCAGAGC | CGGCTGACTCGTTCTCCT |
| Ascl2 | GAGAGCTAAGCCCGATGGA | TCAGTAGCCCCCTAACCAAC |
| Alpi | GCTCAAAGAGGCCCATGA | ATGATCAGAACCTGGTGCAA |
| Atoh1 | TCCCTGAAAACTGAGACAACC | GCTAACAACGATCACCACAGA |
| Defa24 | AGGACCAGGCTGTGTCTGTC | TCTTCCTTTGCAGCCTCTTG |
| Chga | CGATCCAGAAAGATGATGGTC | CGGAAGCCTCTGTCTTTCC |
| Bmi1 | GCAGAAGTTTTGGGAACCCTG | AGTGCCTCAAACGCACTCTC |
| Hopx | GGAGGAGCAGACGCAGAAAT | AGCAGGACAGCAAAACAATG |
| Olfm4 | AGTGACCTTGTGCCTGCC | CACGCCACCATGACTACA |
