# Supplementary figures and images for: Wnt Signaling Mediates the Aging-Induced Differentiation Impairment of Intestinal Stem Cells
Source: Stem Cell Rev. 2019 Feb 21;15(3):448–55. doi: 10.1007/s12015-019-09880-9 (PMC6534527; doi:10.1007/s12015-019-09880-9)

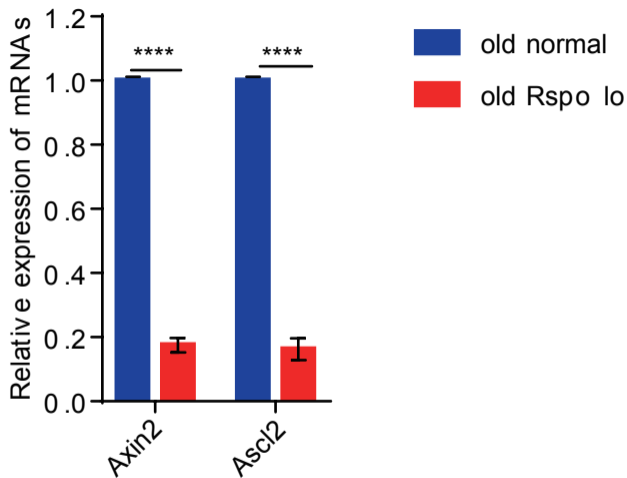

Supplement: Supplementary file 2 — Reduction in R-spondin-1 exposure lowers Wnt signaling activity in cultured ISCs. Freshly isolated crypts from 24 months old mice were plated at a density of 200 crypts per well. mRNA expression of Wnt target genes was analyzed in crypts cultured for 7 days. mRNA expression of genes was normalized to beta-actin with the expression level of each gene in crypts cultured under normal condition set to 1 (n = 3 independent experiments). Data are displayed as mean ± SEM. *, P < 0.05; **, P < 0.01; ****, P < 0.0001. ns, not significant. Unpaired two tailed Student’s t test was used. (PDF 837 kb) [file 12015_2019_9880_MOESM2_ESM.pdf]

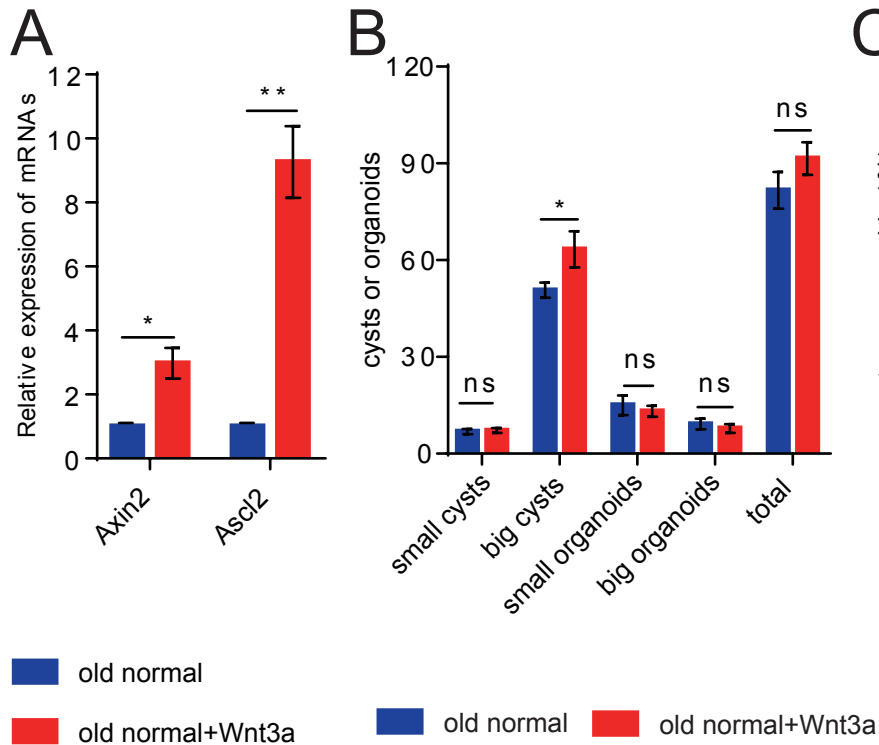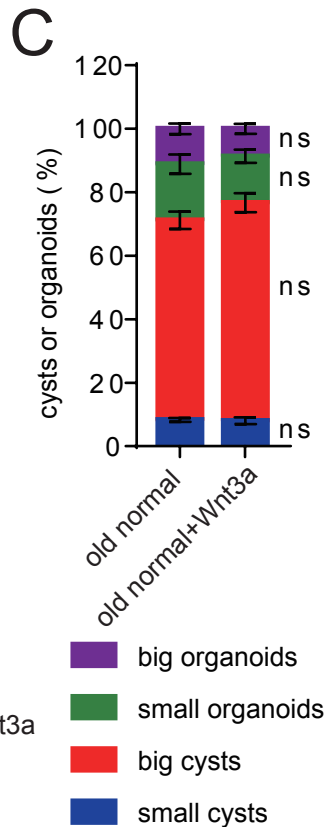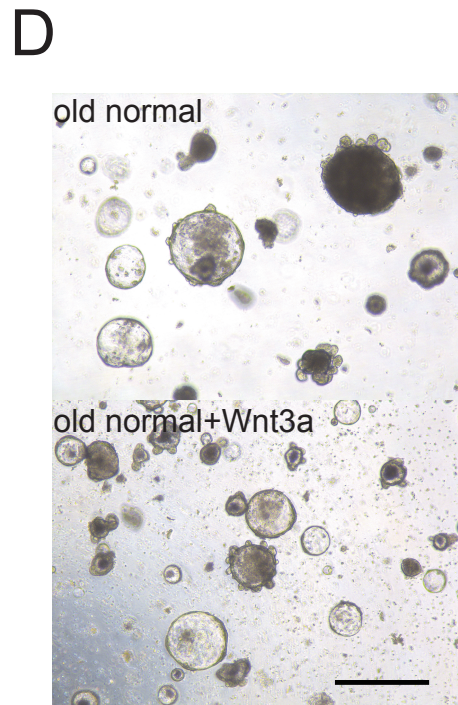

Supplement: Supplementary file 3 — The effect of addition of Wnt3a on old ISCs during primary culture. Freshly isolated crypts from 24 months old mice were plated at a density of 200 crypts per well. Wnt3a was added at a concentration of 100 ng/ml. (A) mRNA expression of Wnt target genes was analyzed in crypts cultured for 7 days. mRNA expression of genes was normalized to beta-actin with the expression level of each gene in crypts cultured under normal condition set to 1 (n = 3 independent experiments). (B-D) Absolute number (B), percentage (C) of grown out structures, and representative pictures (D) on day 7 after primary plating in indicated groups. Results show data from one representative experiment out of 2 independent experiments; n = 3 mice per group. Small cysts: diametres≤70 μm; big cysts: diameters>70 μm; small organoids: with crypt-villus architectures, budding number ≤ 3; big organoids: with crypt-villus architectures, budding number > 3. Data are displayed as mean ± SEM. *, P < 0.05; **, P < 0.01; ***, P < 0.001; ****, P < 0.0001. ns, not significant. Unpaired two tailed Student’s t test was used analysis was used. Scale bar: 200 μm. (PDF 11363 kb) [file 12015_2019_9880_MOESM3_ESM.pdf]
